# Tannin–Mn coordination polymer coated carbon quantum dots nanocomposite for fluorescence and magnetic resonance bimodal imaging

**DOI:** 10.1007/s10856-021-06629-0

**Published:** 2022-01-24

**Authors:** Weibing Xu, Jia Zhang, Zhijie Yang, Minzhi Zhao, Haitao Long, Qingfeng Wu, Fang Nian

**Affiliations:** 1 College of Science, Gansu Agricultural University, Lanzhou, 730000 China; 2 College of Life Science, Gansu Agricultural University, Lanzhou, 730000 China; 3 Institute of Modern Physics, Chinese Academy of Sciences, Lanzhou, 730000 China

## Abstract

The MR/FI bimodal imaging has attracted widely studied due to combining the advantages of MRI and FI can bridge gaps in sensitivity and depth between these two modalities. Herein, a novel MR/FI bimodal imaging probe is facile fabricated by coating the Mn-phenolic coordination polymer on the surface of the carbon quantum dots. The structure of the as-prepared nanocomposite probe is carefully validated via SEM, TEM, and XPS. The content of Mn^2+^ is calculated through the EDS and TGA. The quantum yield (QY) and emission wavelength of the probe are about 7.24% and 490 nm, respectively. The longitudinal r1 value (2.43 mM^−1^ s^−1^) with low r2/r1 (4.45) of the probe is obtained. Subsequently, fluorescence and MR imaging are performed. The metabolic pathways in vivo are inferred by studying the bio-distribution of the probe in major organs. Thus, these results indicate that probe would be an excellent dual-modal imaging probe for enhanced MR imaging and fluorescence imaging.

**Figure:**
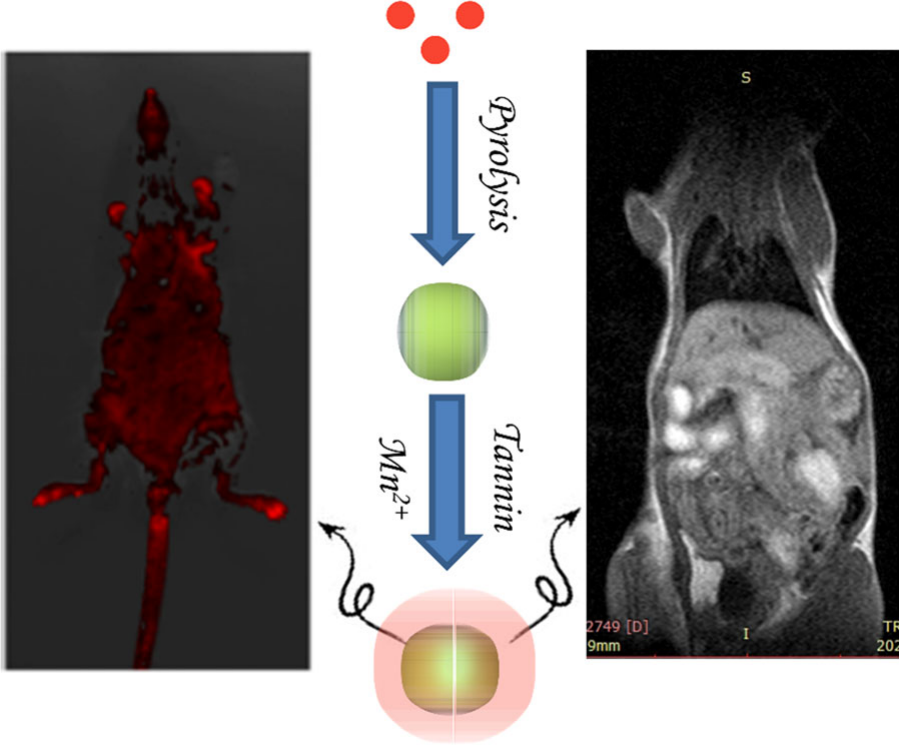
MR/FI bimodal imaging probe is built via in-situ coated Mn-phenolic coordination polymer on the surface of the carbon quantum dots. The in vitro and vivo image property of the probe is evaluated.

MR/FI bimodal imaging probe is built via in-situ coated Mn-phenolic coordination polymer on the surface of the carbon quantum dots. The in vitro and vivo image property of the probe is evaluated.

## Introduction

The poor sensitivity restricts the application of magnetic resonance imaging (MRI) for detecting small tumor nodules [1–3]. Many methods are used to improve the sensitivity of MRI contrast agents. Among them, the introduction of fluorescence groups into the MRI contrast agents to construction of dual-model imaging probe is considered an important way, which will combine the advantages of tissue deep penetration of MRI with the advantages of high sensitivity of fluorescence imaging to overcome their shortcomings and achieve complementary advantages [4–10]. Fluorescent materials commonly used in MRI-FI dual-mode imaging probes include carbon quantum dots (CDs), organic dyes, and metal compounds. Due to the ease of surface functionalization, favorable optical properties, good biocompatibility, excellent water solubility, and low toxicity along with green synthesis, CDs are becoming a superior framework to construct MRI-FI bimodal imaging probes [11–18]. For instance, the synthesis of Gd-dopped CDs for fluorescence/MR imaging by the low temperature pyrolysis of the precursors containing Gd and carbon is reported [19, 20] The longitudinal relaxation rate (r1) and the quantum yield (QY) of the resultant Gd-doped CDs is around 5.5–6.4 mM^−1^ s^−1^ and 2.6–8.9%, respectively. However, it is well known that gadolinium ions (Gd^3+^) are highly toxic because they inhibit calcium channels, induce change in intracellular reactive oxygen species levels, and cause cardiovascular and neurologic toxicity [21, 22] Unlike the lanthanides including Gd, the non-lanthanide metal Mn is a natural cellular consistent, and usually acts as a cofactor for various enzymes and receptors [23]. For several years, the paramagnetic Mn(II) has been at the forefront of contrast agent research as it is believed to be one of the most promising alternatives to Gd(III) [24]. Various Mn(II) complex and composites are fabricated and to be used as MRI contrast agents [24, 25]. For instance, the Gyula Tircsó research group designed and fabricated many kinds of Mn(II)-based MRI contrast agent [26]. However, multi-step process and time-consuming are needed in the process of preparing these small molecular complexes of Mn(II). In addition, in-situ Mn-dopped CDs composites have been reported in many literatures due to the facile fabrication process [27]. However, a large number of metal ions are deeply embedded in the quantum dot, and these metal ions cannot contact the water molecules in the environment because of embedding, so they cannot play the MRI performance [28]. A small number of metal ions bind to the surface of the CDs in Mn-dopped CDs composite, thus giving rise to MRI properties. Therefore, it is believed that increasing Mn ions loaded on the surface of CDs is expected to improve the MRI performance of the composites. Tannic acid (TA) as a type of natural polyphenols exhibits excellent metal chelating ability. The di-galloyl groups rich in TA can serve as chelating sites for multivalent metal ions (such as Mn^2+^) thus to induce rapid self-crosslinking of TA within minutes in water [29]. The TA-metal network could be reform on various substrates to form a conformal coating [30]. This simple and convenient particle surface functionalization technology provides good inspiration for building an MRI-fluorescent dual-mode imaging probe based on manganese ions.

Herein, the tannic acid chelated with Mn^2+^ ions forming shell coating on the surface of blue fluorescent emissive carbon quantum dots (BCQD) to construct novel MR/FI bimodal imaging probe. Compare to the free BCQD, the maximum excitation and emission wavelengths showed an obvious red shift phenomenon after the introduction of tannin-Mn coating on the surface of the BCQD. The content of Mn^2+^ is calculated via the EDS and TGA. The quantum yield of the bimodal probe (QY) is detected to be about 7.24%. The structure of the as-prepared probe is verified in. detail. Afterward, the in vitro fluorescence and MR imaging are performed. The longitudinal r1 value and the ratio of r2/r1 of the probe are calculated. The metabolic pathways in vivo are inferred by studying the bio-distribution of the probe in major organs. Thus, these results indicate that probe would be an excellent dual-modal imaging probe for enhanced MR imaging and fluorescence imaging.

## Expermental

### Chemicals

Tannic acid (TA), MnCl_2_·4H_2_O (≥99%), diammonium citrate (≥99%), and MOPS (≥99%) were purchased from Shanghai Aladdin Bio-chem Technology Co. Ltd. DMSO (≥99.5%) were purchased from Jiangsu Honghui Chemical Co. Ltd. Urea (≥99%) were purchased from Shanghai Yuanye Bio-technology Co. Ltd. Ethyl acetate (≥99.5%) were purchased from Tianjin Lianlongbohua Pharmaceutical Chemistry Co., Ltd. All these chemicals were used without further purification.

### Synthesis of BCQD

0.75 g citric acid and 1.0 g urea (molar ratio 1:5) were dispersed in 30 ml deionized water. The mixture was transferred into 50 mL tetrafluoroethylene and sealed in a stainless-steel autoclave. The autoclave was heated at 180 °C for 7 h. After the mixture cooling to room temperature, the mixture passed through a 0.22 μm injection filter to remove large lumps. Then, it was further purified by dialysis with dialysis bag (M_w_ = 3500) for 48 hours, and the solid samples were obtained by freeze drying and it was named BCQD.

### Synthesis of BCQD@Mn composite

**Scheme 1:**
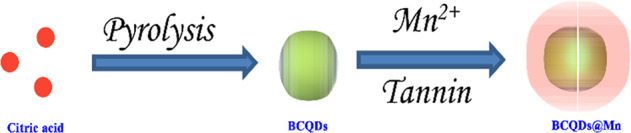
Schematic illustration for fabrication of BCQD@Mn composite

The 50 mg sample was dissolved in 4.9 mL of deionized water with the assistance of ultrasound. Then, 50 μl MnCl_2_·4H_2_O, 50 μl tannic acid (TA) were added into the above solution (final concentration: MnCl_2_·4H_2_O: 0.1 mg/ml, TA: 0.4 mg/ml, BCQD: 10 mg/mL). The solution was vigorously mixed by a vortex mixer for 10 s immediately. The pH of this solution was subsequently raised by adding 0.5 ml of MOPS buffer (20 mM, pH 7.4). The excess TA and MnCl_2_ was removed by dialysis for 48 h. The solution after dialysis was lyophilized to obtain a black powder, named BCQD@Mn. The typical fabrication process was exhibited in Scheme 1.

### Characterization

Using Bruker IFS66v/s IR spectrometer (Bruker, Karlsruhe, Germany) was used to analyze the function group of the sample in the range of 400–4,000 cm^−1^ with the resolution of 4 cm^−1^. The morphologies of BCQD@Mn were characterized using a JEM-1200 EX/S transmission electron microscopy (TEM) (JEOL, Tokyo, Japan) and an S-4800 field emission scanning electron microscopy (SEM) (HITACHI, Tokyo, Japan). BCQD@Mn was measured by TU-1901 dual-beam UV-vis spectrophotometer. The surface elements were measured by X-ray photoelectron spectroscopy (XPS) with VG ESCALAB220i-XL spectrometer.

### Quantum yield

The fluorescence quantum yield (φ) of BCQD@Mn was calculated by using Quinine sulfate solution as a standard. Quinine dissolved in 0.5 M H_2_SO_4_ was used as a standard with a known φ value of 54% at 345 nm excitation wavelength. The φ of BCQD@Mn was calculated by comparing the UV-vis absorbance values and the integrated fluorescence intensities (λ_ex_ = 360 nm) of the BCQD@Mn with those of quinine sulfate solution. The absorbance values of all solutions were maintained under 0.05 to minimize self-absorption. Eq. 1 was used for the calculation of φ of BCQD@Mn.

$$\varphi = \varphi _s \times \frac{I}{{I_s}} \times \frac{{A_s}}{A} \times \frac{{\eta ^2}}{{\eta _s^2}}$$

where φ is fluorescence quantum yield. I is the integrated fluorescence intensity, A is the UV-Vis absorbance, and ƞ is the refractive index of the solvent for BCQD@Mn suspension solution as water (*ƞ* = 1.33) and 0.5 M H_2_SO_4_ in water (*ƞ* = 1.76).

### Cytotoxicity assay

Typically, HepG2 cells (5 × 10^3^ cells/well) were seeded into a 96-well plate and further incubated in RPMI1640 containing FBS (10%) at 37 °C for 24 h. Following overnight incubation, the BCQD@Mn composite was added at different concentrations for 48 h. After incubation, the MTT reagent (20 μL, 5 mg/ml) was added into each well of the 96-well culture plate, and cells were incubated for another 4 h at 37 °C in an atmosphere of 5% CO_2_. Then the medium was taken out and 150 μL DMSO was added to each well. Cell viability was assessed by the optical density (OD) value at a wavelength of 490 nm using an enzyme-labeled instrument.

### In vitro and vivo fluorescence imaging

The HepG2 cells (2 × 10^5^ cells/well) were seeded into six-well plates, cultured for 24 h, and treated with BCQD@Mn composite for 48 h. After treatment, the culture medium was discarded, and cells were washed three times with PBS. Then the cells were washed three times with PBS for 7.0 min each time and imaged by fluorescence microscope. In vivo fluorescence imaging experiments were conducted in Ultra-sensitive fluorescence imaging system for small animals (VISQUE In-vivo Smart-LF, Viewers). The BCQD@Mn composite was dissolved in PBS at a concentration of 20 mg/ml. Healthy male Kunming mice (Experimental Animal Canter of Lanzhou University, 20 ± 2 g) were randomly divided into two groups (*n* = 3) and were given of the BCQD@Mn composite one time *via* tail vein (20 µL injection volume, 20 g/kg), control group received PBS. All animals were sacrificed after seven days. Major organs (Heart, liver, spleen, lung, kidneys) were removed, washed with PBS, and fixed in 4% formaldehyde, embedded in paraffin, sectioned, and then stained with hematoxylin and eosin for histological analysis. Mice were intravenously injected with the composite solution (10 mg·kg^−1^) and fluorescence imaging was conducted at 0.5, 1, 2, 4, and 24 h post injection. Ex vivo fluorescence imaging of major organs was also conducted.

### In vitro and in vivo MRI performance

In vitro MRI experiments were conducted in a 0.5 T nuclear magnetic resonance analyzer (MesoMR23-060H-I, Suzhou Niumag Analytical Instrument Co., China). The MRI samples were prepared by drawing BCQD@Mn composite solutions at different Mn (II) concentrations into 1.0 mL disposable poly (propylene) syringes. For the T1 measurement, inversion-recovery fast spin-echo sequence with eight inversion times (TI; ranging from 10 ms to 4 s) was employed. Meanwhile, for the T2 measurement, multiclade multi-echo sequence was employed. [Others parameters: TR = 6 s (for T1) and 2 s (for T2); matrix size = 128 × 128; field of view = 40 × 40 mm; slice thickness = 4 mm). The r1 and r2 values were calculated from the slop of curve-fitting result of 1/T1 and 1/T2 (s^−1^) versus the Mn(II) concentration (mM). for in vivo MRI, healthy male Kunming mice (20 ± 2 g,) were also randomly divided into two groups (*n* = 3). The animal was anesthetized using chloral hydrate (10 wt%, 80 μL). 20 μL of the Mn(II) composite (40 μg mL^−1^) solution was injected into the animal one time via the tail vein. MRI was performed at the pre-injection and 0.5, 1.0, 4.0, and 24 h after injection. The T1-weighted coronal MRI was acquired using a MesoMR23-060H-I 0.5 T MRI scanner with the following imaging parameters: repetition time, 330 ms; echo time, 18.2 ms; field of view, 100 × 100 mm^2^; and slice thickness, 3.0 mm.

## Results

### Structural characterization

**Fig. 1:**
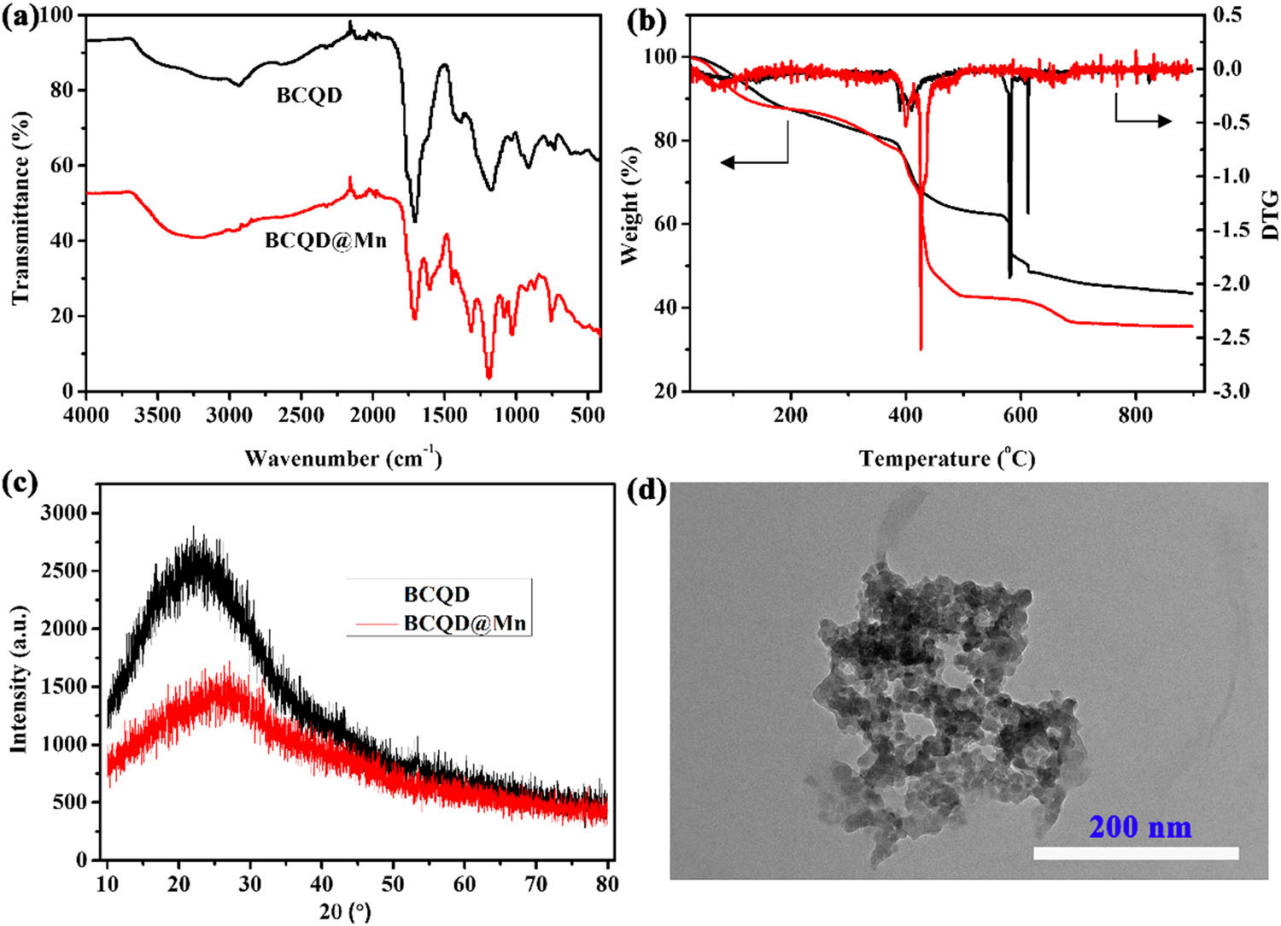
IR spectrum (**a**), TGA and DTG curves (**b**) and XRD pattern (**c**) of BCQD and BCQD@Mn; (**d**) TME image of the BCQD@Mn composite

**Fig. 2:**
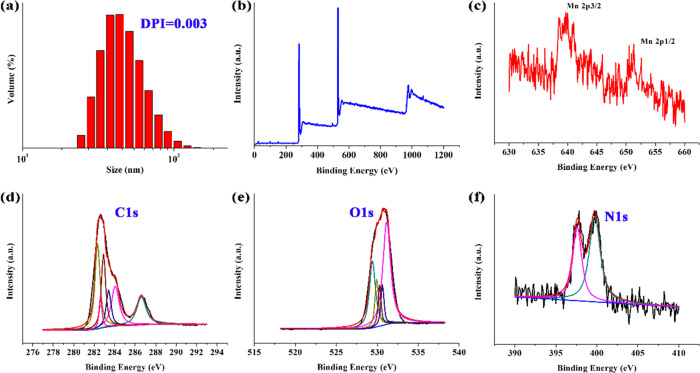
DLS curve (**a**) and XPS spectra of the BCQDs@Mn composite: (**b**) survey spectra, (**c**) Mn 2p, (**d**) C 1s, (**e**) O 1s, (**f**) N 1s

The detailed preparation process is described in Scheme 1. The preparation of the composite underwent two steps including pyrolysis and coating. The UV-Vis absorption curves of BCQD and BCQD@Mn samples are displayed in Fig. S1a. Obvious absorption peaks in the near-ultraviolet region of 190–210 nm can be observed for the BCQD. As for the BCQD@Mn, two significant peaks are found and the peak’s center locates at 210 and 280 nm, respectively. The fluorescence emission spectra of BCQD at different excitation wavelengths and concentrations are exhibited in Fig. S1b, c. The relative intensity increases first and then decreases with the increase of excitation wavelength. The maximum excitation and emission wavelength is 350 and 450 nm, respectively. In addition, a good dependence relationship is observed between the intensity and concentration. The relationship between excitation wavelength and concentration and emission intensity of BCQD@Mn composite is like that of the BCQD (Fig. S1d, e). The maximum excitation and emission wavelengths of the BCQD@Mn composite is observed at 390 nm and 490 nm (Fig. S1f), respectively. The fluorescence quantum yields in PBS are determined to be 7.24% according to the corresponding formula and quinine sulfates are used as a reference [31]. The FTIR spectrum of the BCQD and BCQD@Mn complex is shown in Fig. 1a. A broad absorption band can be seen at 3400 and 1740 cm^−1^ for both samples, which is assigned to the characteristic stretching vibration of –OH and C=O groups [32, 33]. The peaks located at 757 and 1314 cm^−1^ can be ascribed to the stretching vibration of C–H in both samples. The characteristic peak at 1030, 1082, and 1189 cm^−1^ can be assigned to the stretching vibration of C–O in both samples [34]. The TGA and DTG curves of the BCQD and BCQD@Mn complex are presented in Fig. 1b. Three weight loss steps are found for the BCQD. The first one located in the temperature range of 100–400 °C is assigned to the oxidation of functional groups in the sample [35]. The second sharp process of weight loss between 400 and 600 °C is ascribed to the carbonization of skeletons in the BCQD [36]. The third bluff type of weightlessness is in the temperature range of 600 to 700 °C. There are three exothermic peaks on the DTG curve, and the largest one is between 600 and 700 °C. For the BCQD@Mn, the weightlessness process is basically similar to that of the BCQD. The XRD patterns of both samples showed a diffraction peak at 22.5°, which is attributed to the graphitized structure of carbon. The intensity decreases after the introduction of TA-Mn coating on the surface of BCQD. This may be related to reducing the crystallinity without affecting its structure. The morphology of BCQD@Mn composite is studied by TEM (Fig. 1d). Many nanoparticles with regular spherical structures have been observed, which are cross-linked with each other due to agglomeration. Meanwhile, the size is about 20 nm according to the statistics. This indicates that the structure of the BCQD is kept very well in the process of introducing TA-Mn coating. In addition, DLS is also used to monitor the preparation of the BCQD@Mn (Fig. 2a). It can be found that the average hydrodynamic diameter is calculated to be 64.41 ± 16.67 nm from a DLS pattern. The PDI value is as low as 0.003, indicating that the high granular uniformity of the composite. The value of D10, D50, and D90 is around 28.68 nm, 41.54 nm, and 65.76 nm, respectively. This means that the average size is less than 28.68 nm, 41.54 nm and 65.76 nm particles account for 10%, 50%, and 90% of all particles. This value is larger than the average size estimated from TEM. This may be ascribed to a large number of hydrated water molecules around the composite NPs in the DLS test procession. The successful formation of BCQD@Mn is further verified by XPS spectra. The survey spectrum confirmed that BCQD@Mn is mainly composed of C, N, O, and Mn (Fig. 2b). The corresponding characteristic peaks for C 1s, N 1s, O 1s, and Mn 2p are observed at 285.6, 399.32, 531.9, and 640 eV, respectively. Moreover, the content of all elements from the XPS results is summarized and inserted in Fig. 2b. As expected, XPS results confirmed the presence of Mn in the product with 0.34 At% (Table S1). The high-resolution spectra of Mn 2p (Fig. 2c) present two peaks at 652.6 and 642.1 eV, which agreed with the spectra of Mn (2p1/2) and Mn (2p3/2) [37]. The high-resolution XPS spectra of C 1s (Fig. 2d) revealed the presence of sp3 and sp2 hybridized carbon atoms and carboxylate/hydroxyl moieties in the composite [38]. The fitted peaks in N 1s spectra suggest the C–N, C=N, and graphitic-N content are involved in the composite. As for O 1s, the fitted peaks correspond to C–O and C=O. The energy-dispersive X-ray spectroscopy (EDS) analysis demonstrated the distribution of Mn, O, C, and N elements in the BCQD@Mn composite (Fig. S2a). The relative content in mass percentage of the C, N, O, and Mn is around 67.71%, 3.87%, 27.48%, and 0.94 At % (Table S2), respectively. The elemental mapping (Fig. S2b–f) revealed the four elements are uniformly distributed in the composite. This result implied that the TA-Mn coating is fully confined within the surface of BCQD during the reaction process, and all components are strongly coupled together in the composite. The stability of the BCQD and BCQD@Mn composite is inspected *via* the change in particle size and potential with immersion time (Fig. S3a, b). The average particle sizes and PDI of BCQD and BCQD@Mn composite is detected to be 120 nm, 150 nm, and 0.461, 0.409 on the first day. Both numerical of the BCQD@Mn composite increase up to 163 nm and 0.507 as the time extends to fifth day. Although the size of BCQD shows insignificant change over time, the PDI value increased by 0.523. The slight trend of both values even immersed in PBS solution for 120 h in PBS indicate good stability performance of both samples. The Zeta potential values of BCQD@Mn composite are −15.4 and −16.3 mV for the first and fifth day, respectively. This value is significantly higher than −25.3 and −26.1 mV of BCQD at the same time. this may be related to the introduction of the TA-Mn layer on the surface of the BCQD. This further reflects the excellent stability of the BCQD@Mn composite in solution state. The content of Mn in the BQCD@Mn is further estimated via the atomic absorption spectrum. Firstly, a calibration curve is established by using the function relationship between the concentration of different manganese ions and the absorption intensity (Fig. S3c). A good linear correlation can be found between intensity and the concentration of Mn^2+^ with a correlation coefficient of R^2^ = 0.99944. The BCQD@Mn (1.0 mg) is formulated into a 10 mL solution in deionized water. The content of Mn in the composite is calculated by comparing the absorption intensity of the above solution to the calibration curve. The content of Mn is about 0.22 mM in the BCQD@Mn composite solution.

### Cytotoxicity and fluorescence bioimaging

**Fig. 3:**
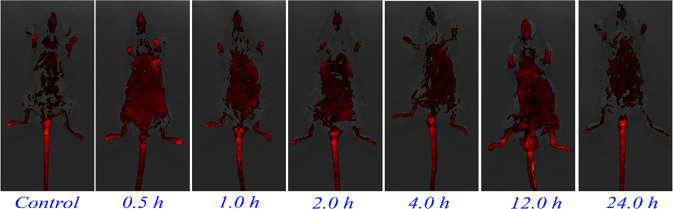
In vivo whole-body fluorescence images of mice with intravenous injection of the BCQD@Mn at different time points (*n* = 3)

**Fig. 4:**
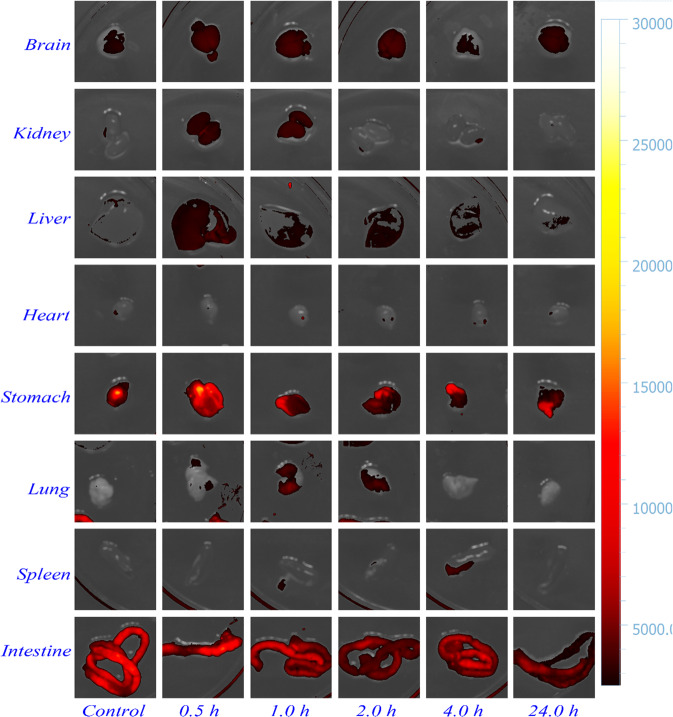
Real-time ex vivo imaging of mice with intravenous injection of the BCQD@Mn composite at different time points (*n* = 3)

It is believed that low toxicity is emerging as the focus of designing metal complexes for the diagnosis of cancers. MTT assay is employed to evaluate the cytotoxicity of BCQD@Mn composite toward HepG2 cells. As shown in Fig. S3d, the cell viability is over 90% even when the concentration of BCQD@Mn reaches 100 µg mL^−1^ (equivalent Mn concentration is 0.06 mM) for 48 h incubation, indicating the safety and negligible cytotoxicity of the composite towards HepG2 cells. This may be ascribed to the strong Mn-TA chelation effectively restraints the leakage of Mn^2+^ into the surroundings. The feasibility of the BCQD@Mn composite for in vitro luminescent cell imaging in HepG2 cancer cells after 24 h-incubation is conducted by fluorescence microscope. As illustrated in Fig. S4a, d, g, the bright-field image of BCQD@Mn with different amount demonstrated the morphological integrity of cells, validating their biocompatibility. The fluorescence of the cells is distinctly enhanced with the amount of BCQD@Mn (Fig. S4b, e, h), suggesting a dosage-dependent fluorescence enhancement relationship. It should be noted that the BCQD@Mn are observed to localize in the whole cell area, weak emission signals from the nucleus and strong ones from the cytoplasm (Fig. S4c, f, i), indicating excellent cell membrane permeability. The tubes fluorescence images of BCQD@Mn composite solutions are obtained under four different excitation wavelengths (Fig. S5a–d). The solution only exhibits good fluorescence properties at the excitation wavelength of 390–490 nm. Afterward, the nanoplatform as a in vivo fluorescent imaging agent is explored. The whole-body fluorescence images of mice are taken at different time points after the tail vein injection of BCQD@Mn composite (Fig. 3). It can be found that the fluorescence of BCQD@Mn composite could efficiently penetrate mice skin and tissues. The mice injected with PBS exhibits the weakest fluorescence, which is derived from the spontaneous fluorescence of mice under the selected excitation wavelength. No animal showed any signs of acute toxicological responses during the experiments. In order to further explore the metabolic pathway of the probe, fluorescence imaging of the main organs at different time points is performed (Fig. 4). The mice are sacrificed at different time points (24, 4, 2, 1, and 0.5 h) to obtain organs imaging at the same time. The fluorescence intensity of the same dissected organ at different time points is gradually reduced, this is agreeing well with the whole-body fluorescence imaging. Besides, fluorescence signals of the brain, liver, and kidney are always much stronger than these of heart, spleen, and lung. Surprisingly, significant fluorescent signals are observed in the brains of the mice at the 0.5, 1, and 2 h, respectively, suggesting the potential of the BCQD@Mn composite to cross the blood-brain barrier, which may provide a valuable strategy for the theragnostic of some brain diseases through the real-time tracking. In addition, the strong fluorescence signal is detected in the organs of kidney and liver and the fluorescence signal in these two organs basically attenuates synchronously. The above data show that the BCQD@Mn composite can be removed from the body by the kidney system, but may also be metabolized through the liver, and thus into the intestines, in the form of feces to exclude the body. This is confirmed by the variation of fluorescence signals in the intestine over time. Therefore, it can be summarized those two metabolic pathways are found including rapid kidney excretion and liver metabolizing. the above data strongly suggest the BCQD@Mn composite possesses excellent biocompatibility and strong penetrability, which will rapidly enter the whole body of mice with the blood circulation rather than accumulate at the injection site.

### In vitro and in vivo MR imaging

**Fig. 5:**
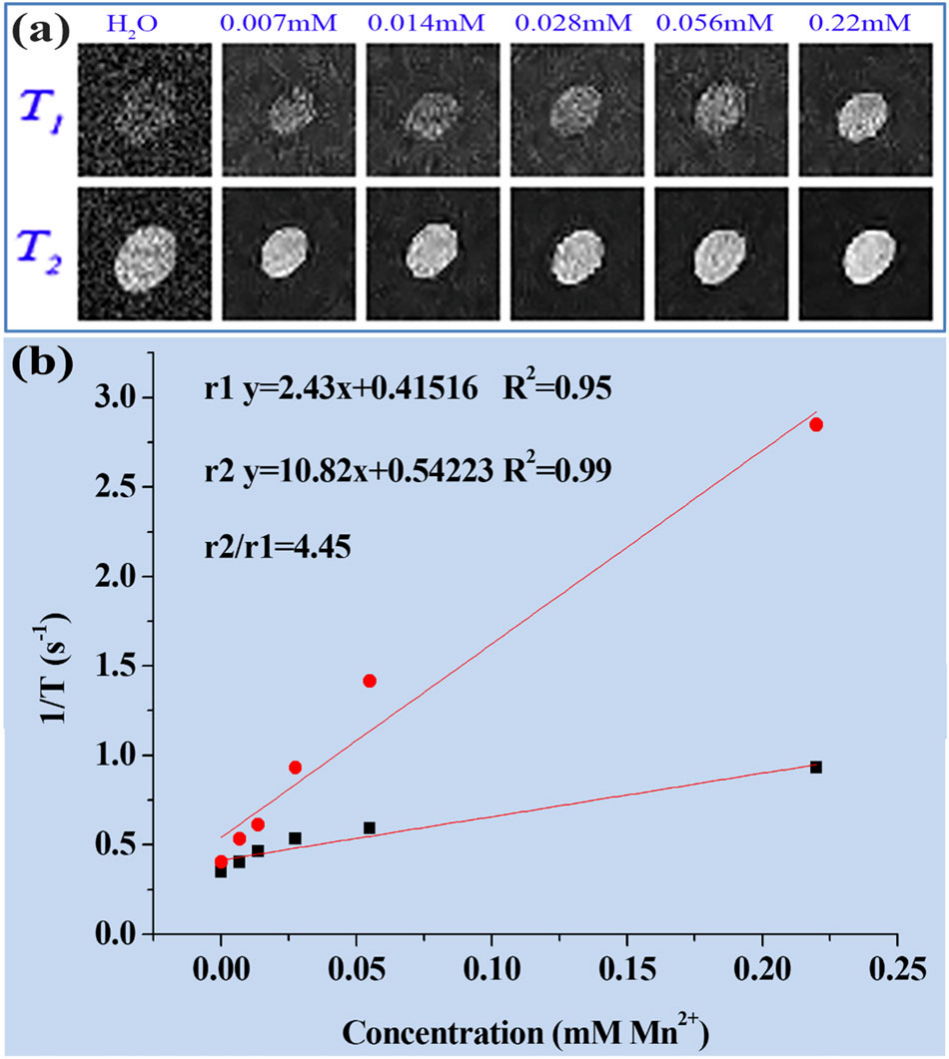
**a** In vitro T1 and T2-weighted MRI images, (**b**) r1 and r2 values of the BCQD@Mn composite at different time points

**Fig. 6:**
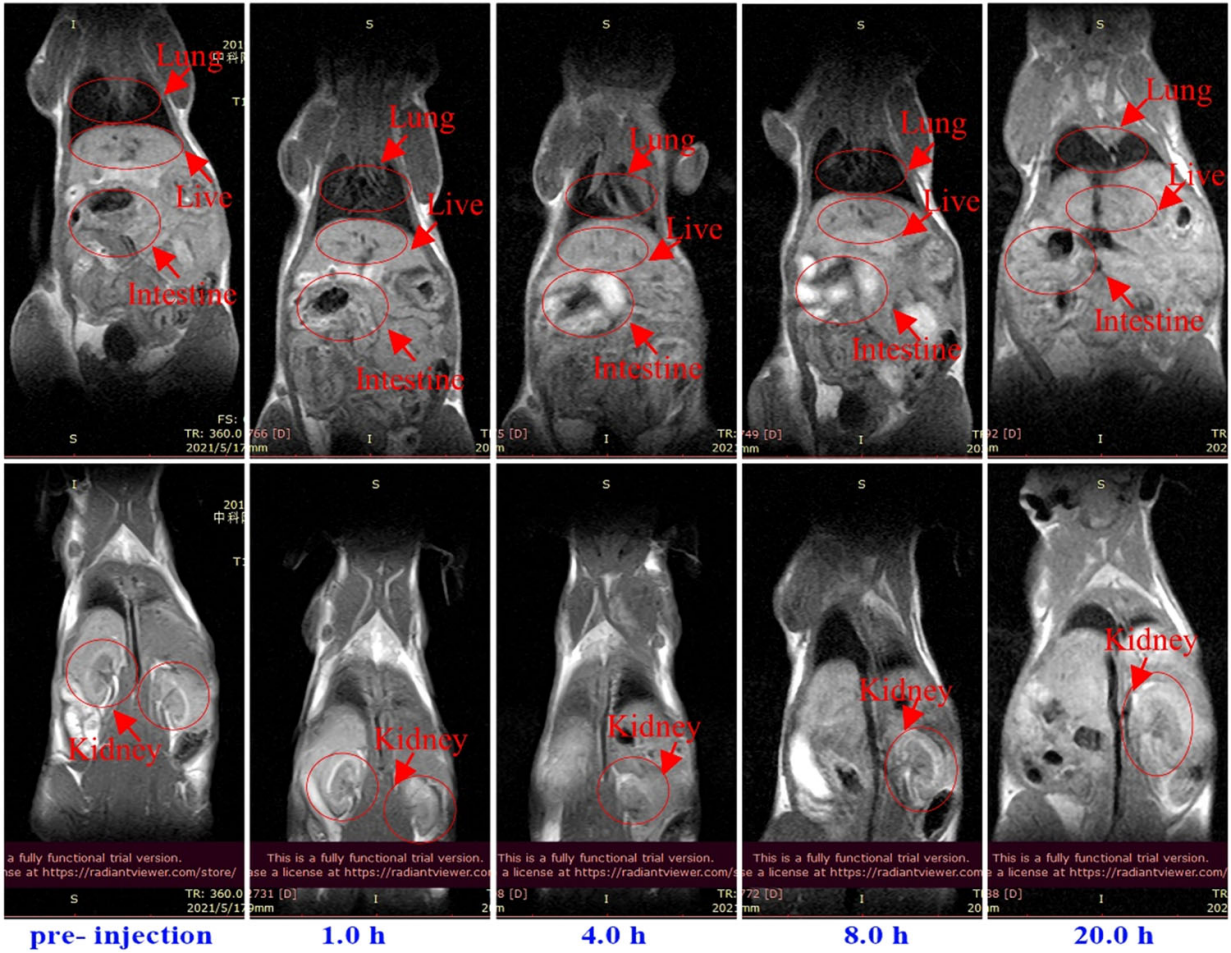
In vivo T1-weighted MRI images of mice with intravenous injection of the BCQD@Mn composite at different time points

Manganese ion (Mn(II)) has been proved to be a safe paramagnetic ion, and MRI contrast agents based on Mn (II) has attracted extensive attention for many years. The performance of BCQD@Mn composite as potential MRI CAs is investigated. The T1 and T2 MRI images are illuminated in Fig. 5a. Although the brightness of both T1 and T2 images increases with the increase of Mn(II) concentration, the improvement degree of T1 images is significantly higher than that of T2 images, indicating the composite gives clearly enhanced MR signals in the in vitro imaging experiments. The relaxivity r1 and r2 derived from the concentration-dependent T1 and T2 measurement is found to be 2.43 and 10.82 mM^−1^ S^−1^ (Fig. 5b), respectively. These results suggest the BCQD@Mn composite could be a good probe for MRI CAs. In vivo, MRI is investigated in normal mice. The time-dependent T1-weighted 2D coronal images of pre- and post injection of BCQD@Mn composite at various time points are shown in Fig. 6. The particles are intravenously injected into the animals. It is clearly observed that the T1 MR signal in the liver and kidneys increases significantly within the 1 h post injection and then decreases as the time further extension. However, the T1 MR signal in the intestine organ gradually increased within 8 h, and then turned weaker over time. After 24 h, signals in most of the organs had subsided to the pre-injection levels, indicating excretion of the particles from the circulation. Interestingly, the signal change in the bladder is found to be small throughout the course of the experiment. Instead, there is a dramatic increase of signals in the liver and intestinal, indicating the liver metabolism is the main metabolic pathway of the BCQD@Mn composite.

## Discussion

During the current decades, the great numerous of Fl-MR dual-mode imaging probs based on Gd^3+^ has been investigated [39]. Nevertheless, most of the currently approved contrast agents based on Gd^3+^ possess long-term toxicity for living imaging. To overcome this problem, the BCQD@Mn nanocomposite is designed with a two-step approach. BCQD are firstly synthesized by one-pot pyrolysis method, and Mn-TA films are coated onto BCQD with rapid layer by layer interactions between TA and MN (II) ions.

The successful synthesis of the BCQD@Mn nanocomposites is proved by several evidences. As shown in the UV-Vis spectra in Fig. S1a, a new peak is found at 280 nm for the BCQD@Mn nanocomposite, indicating the existence of p–π* and n–π* transition in the nanocomposite. This may be attributable to the existence of sp^2^ hybridized carbon atoms [31], suggesting the introduction of the TA-Mn coordination polymer coating on the surface of the BCQD successfully. Similarly, the maximum excitation and emission wavelengths in the fluorescence spectrum (Fig. S1f) show an obvious red shift phenomenon, reaching 390 nm and 490 nm after the surface of the BCQD is coated by tannin-Mn layer. This may be due to the introduction of coordination polymer layers to increase the conjugated structure of the system. In the FTIR spectrum (Fig. 1a), the absorption band at about 1605 and 1442 cm^−1^ in the BCQD@Mn complex is ascribed to the characteristic of the stretching of the benzene ring of TA [40]. This suggests that TA-Mn coordination polymer is successfully introduced on the surface of the BCQD. As for the TGA process (Fig. 1b), the maximum weightlessness process of the BCQD@Mn composite occurs between 400 and 600 °C, which is completely different from the range for BCQD. This may be related to the introduction of TA-Mn coating. Furthermore, the XRD peaks of the synthesized BCQD@Mn nanocomposites are consistent with BCQD (Fig. 1c), and the peaks exhibit slight broadening and uneven peak profiles due to the thick TA-Mn films out of the BCQD cores [41]. The films surrounding the BCQD@Mn nanocomposites NPs can be easily identified in the TEM images (Fig. 1d). The EDS and XPS results (Fig. S2 and Fig. 2b–f) also indicate that Mn (II) has cooperated into this nanocomposite (Tables S1, S2). The test value of EDS is greater than XPS for Mn element, which is related to the XPS can only obtain the surface element content of nanoparticles [42]. Together with the above characterizing result, Mn-TA films are confirmed to be formed through complexation between tannic acid molecule and Mn (II). More importantly, MPN films obviously enhance the biocompatibility and lower cytotoxicity (Fig. S3b) due to the water solubility of tannic acid molecule.

Afterward, the MR/FI bi-modalities imaging performance of the BCQD@Mn nanocomposite is synergistically investigated. The CLSM observes that the HepG2 cells incubated with the nanocomposite show green fluorescence imaging (Fig. S4). The clear and important information about cell morphology and physiological characteristics can be obtained from the visible cell imaging. The MRI property of the as-prepared BCQD@Mn nanocomposite is mainly due to the five unpaired inner electrons of Mn(II) [43]. T1 and T2 weighted images (Fig. 5a) reflect the efficiency of a MRI contrast agent, and the r1 value of BCQD@Mn nanocomposite in vitro is 2.43 mM^−1^ s^−1^ (Fig. 5b). In other word, this reflects that the BCQD@Mn nanocomposite can be served as bio-marker. On this basis, the in, ex vivo fluorescence imaging and in vivo MRI is exhibited in Figs. 3, 4 and 6. It can be seen that the fluorescence is stronger than that of the control group at all post injection time points even 24 h. Not surprisingly, the fluorescence intensity gradually slight decreases as the extension of time. This suggests that the complex has a long residence time in vivo, which would facilitate multiple iterations of imaging after a single injection. However, fluorescence imaging is mainly limited by two aspects: on the one hand, the tissue penetration ability of fluorescence is weak, the fluorescence agents in vivo are difficult to be excited, and the emitted fluorescence is also difficult to be detected. On the other hand, spontaneous fluorescence from biological tissue can cause serious interference to the fluorescence of probes [44]. In view of the possible background interference of whole-body fluorescence signals, the fluorescence signals of ex vivo organs tissues with whole-body MRI signals are compared in Figs. 4 and 6. It can be found that fluorescence signals in the kidneys and liver reaches the highest levels at 1 h of post injection, while MRI signals in both organs improve significantly at the same post injection time compare to the control. Although the fluorescence signals in the liver and kidneys decreased significantly at 1 h of post injection, the signal fluorescence signals in the intestine began to increase significantly from this point on, this indicates that the compound enters the small intestine rapid metabolism through the liver and kidneys. Correspondingly, the exact signal change relationships could be found in intestine T1 MR signal. Figure 6 shows that the in vivo intestine T1 MR signal gradually increased within 8 h post injection. The complementary of fluorescence signals from ex vivo organs and in vivo MRI signals shows that there are two metabolic pathways included in liver and kidneys of the composites in mice. The above results showed that BCQD@Mn nanocomposites possess an excellent MRI and fluorescence di-model imaging probe in vivo. This is very useful for obtaining comprehensive diagnostic information from different images of the same tissue or organ.

## Conclusions

In conclusion, TA-Mn coordination polymer is introduced onto the surface of BCQD by a convenient in-situ synthesis method to successfully fabricate novel MRI-FL di-model image probe. Instead of damaging the fluorescence of quantum dots, the introduction of TA-Mn coating redshifts the emission wavelengths of CQDs, which is more conducive to fluorescence imaging. Interestingly, the BCQD@Mn(II) shows excellent bright orange fluorescence properties with the QY 7.24%, as well as paramagnetic nature with the values of r1 and r2 is about 2.43 and 10.82 mM^−1^ S^−1^. Real-time fluorescence imaging in vivo shows that the probe can easily enter the body of mice and gradually attenuated with time. Fluorescence imaging of the ex vivo organ revealed that the probe has two metabolic pathways in the body, the liver, and the kidneys. The results indicate that probe will be an excellent dual-modal imaging probe for enhanced MR imaging and fluorescence imaging.

## Supplementary Information

Revised supporting information
