# Supplementary material for: Tannin–Mn coordination polymer coated carbon quantum dots nanocomposite for fluorescence and magnetic resonance bimodal imaging
Source: J Mater Sci Mater Med. 2022 Jan 24;33(2):16. doi: 10.1007/s10856-021-06629-0 (PMC8786750; doi:10.1007/s10856-021-06629-0)
Supplement: Supplementary file 1 — Revised supporting information [file 10856_2021_6629_MOESM1_ESM.docx]

Tannin-Mn coordination polymer coated carbon quantum dots composite for fluorescence and magnetic resonance bimodal imaging

Weibing Xu^1,^ *, Jia Zhang^1^, Zhijie Yang^2^, Minzhi Zhao^1^, Haitao Long^1^, Qingfeng Wu^3^, Fang Nian^1,^ *

^1^ College of Science, Gansu Agricultural University, Lanzhou 730000, China.

^2^ College of Life Science, Gansu Agricultural University, Lanzhou 730000, China.

*^3^ Institute of Modern Physics, Chinese Academy of Sciences, Lanzhou 730000, China.*


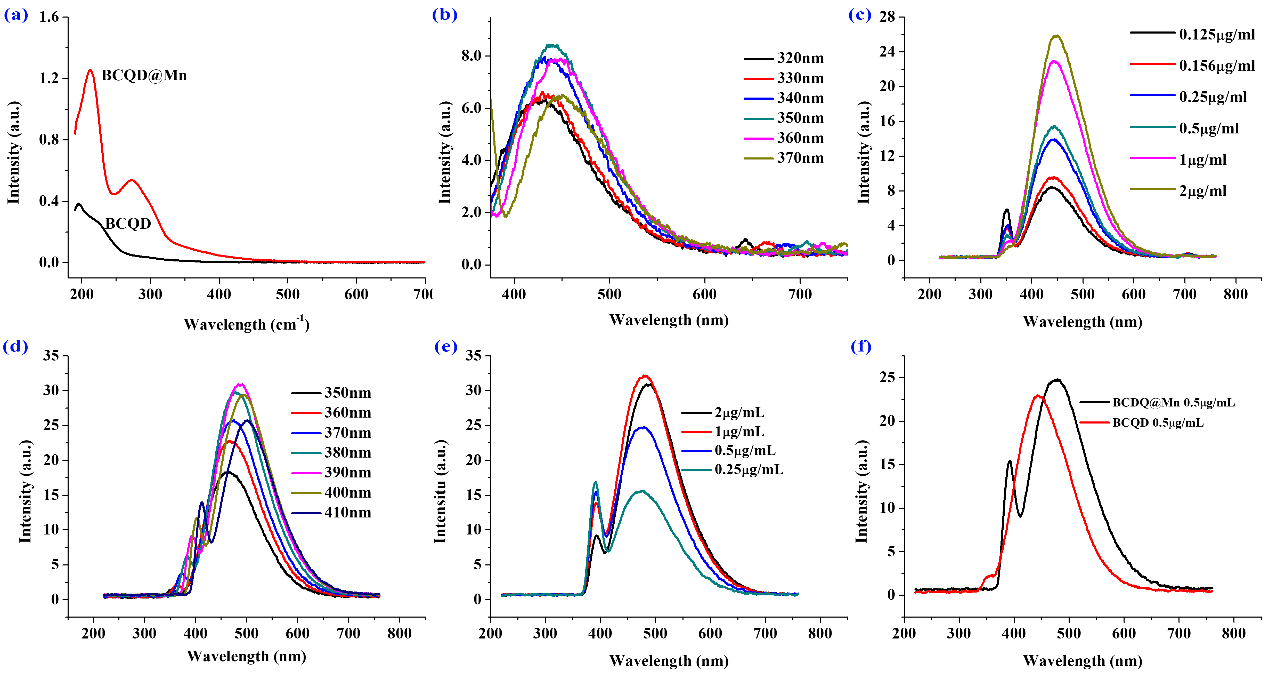


Fig. S1 (a) the UV-Vis spectra of the BCQDs and BCQDs@Mn composite, the fluorescence emission spectra of BCQDs at different excitation wavelength (b) and concentration (c); the fluorescence emission spectra of BCQDs@Mn at different excitation wavelength (d) and concentration (e); the comparison fluorescence emission spectra of BCQDs and BCQDs@Mn (f).


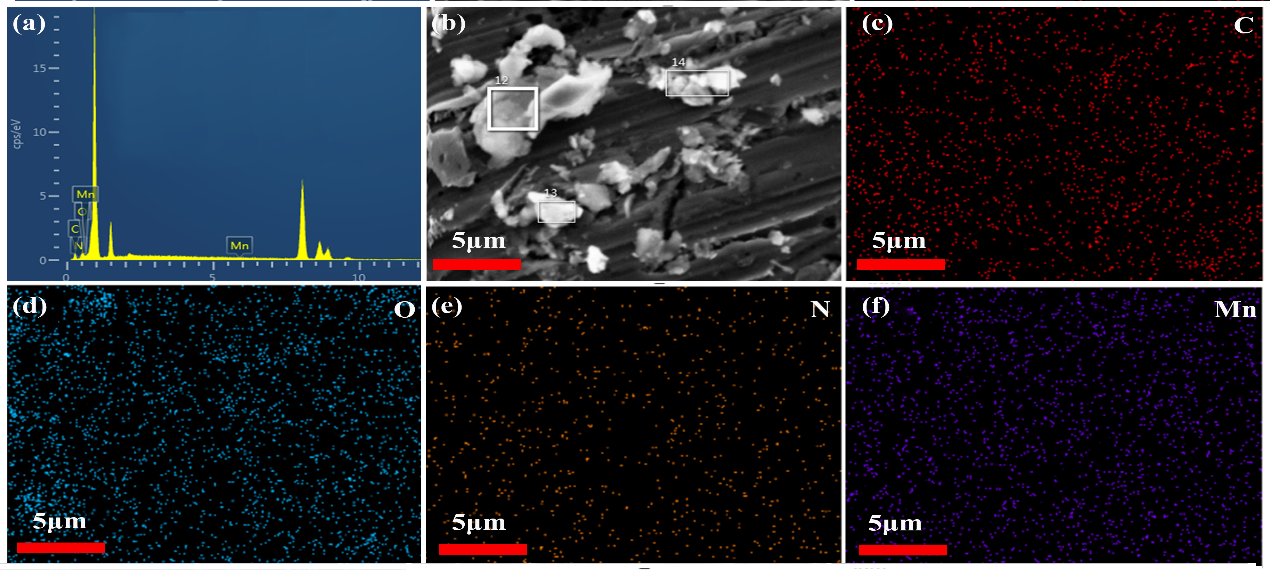


Fig. S2 EDS curve (a) and elements mapping (b-f) of the BCQDs@Mn composite.


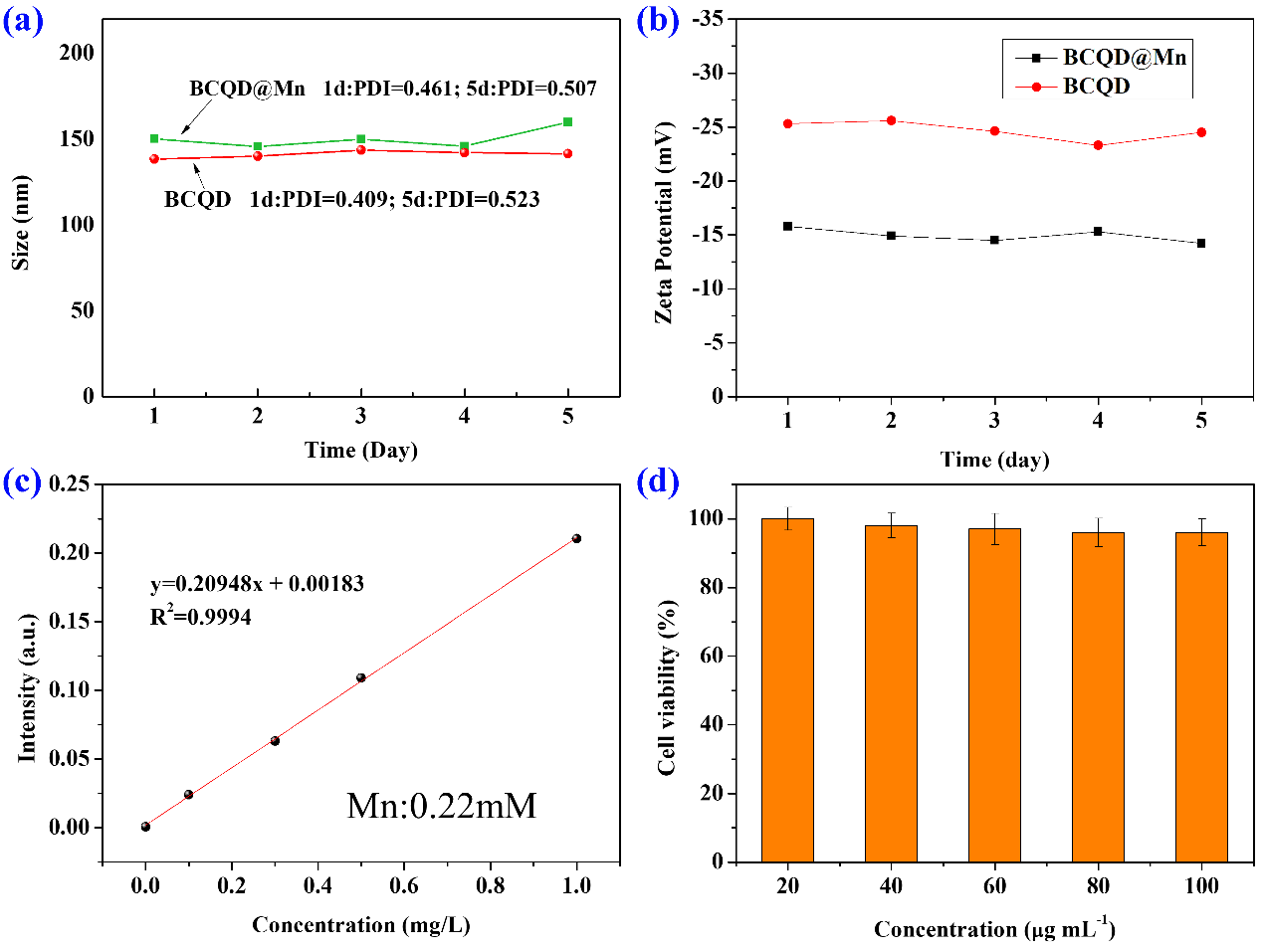


Fig. S3 (a)DLS and Zeta(b) potential of the BCQD and BCQDs@Mn composite for a period of five days, (c) the atomic absorption calibration curve of Mn^2+^ and (d) the cell viability of BCQDs@Mn composite.


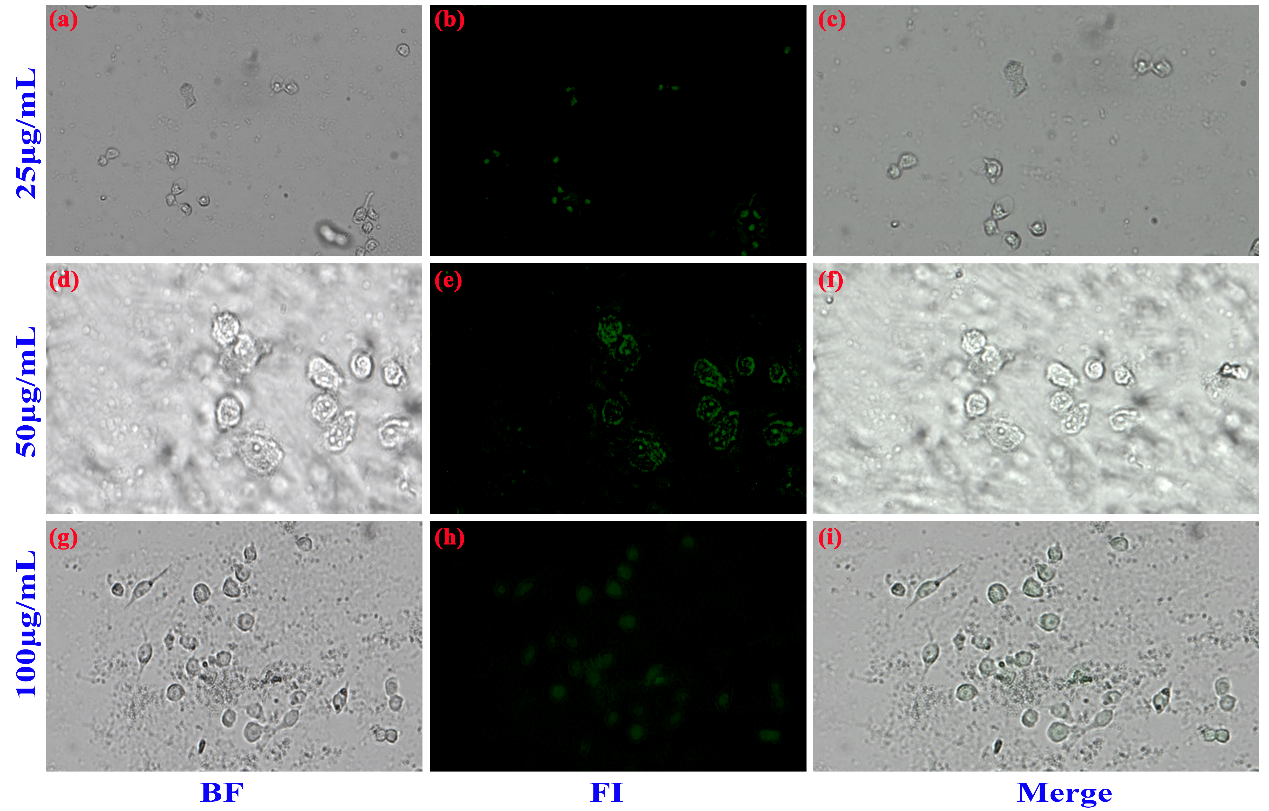


Fig. S4 Fluorescence images of HepG2 cells incubated with 25, 50 and 100 µg mL^-1^ BCQDs@Mn composite for 24 h.


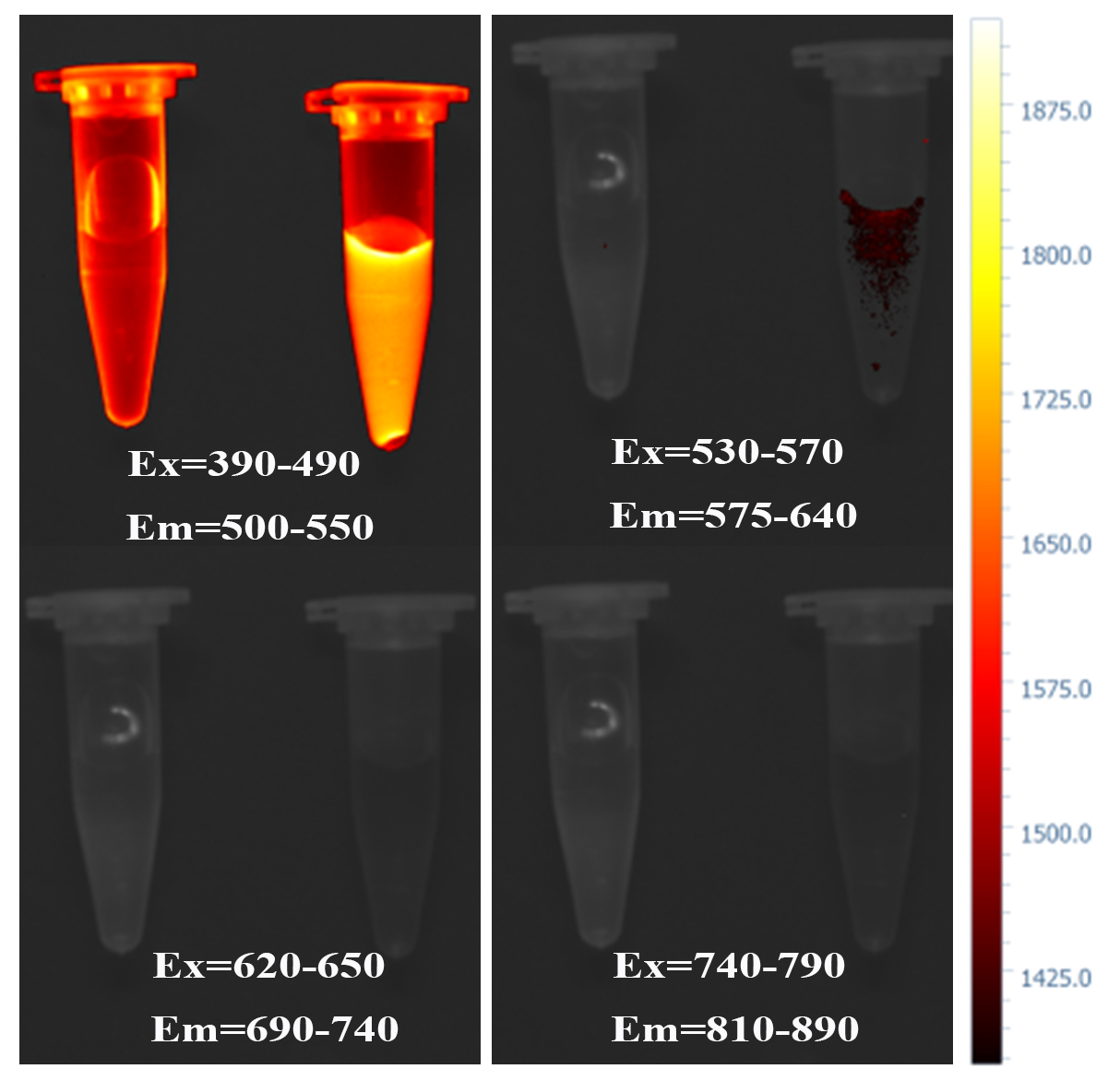


Fig. S5 Tube fluorescence images of BCQDs@Mn composite with 25 µg mL^-1^ at various excitation wavelength

Table S1 the XPS content of C, N, O and Mn elements in the BCQDs@Mn composite

| **Element** | **At%** |
| --- | --- |
| **C** | **14.05%** |
| **N** | **1.26%** |
| **O** | **80.35%** |
| **Mn** | **0.34%** |
| **Total** | **100.00** |

Table S2 the EDS content of C, N, O and Mn elements in the BCQDs@Mn composite

| **Element** | **At%** |
| --- | --- |
| **C** | **27.48** |
| **N** | **3.87** |
| **O** | **67.71** |
| **Mn** | **0.94** |
| **Total** | **100.00** |
